# The Prognostic Impact of Circulating Tumour DNA in Melanoma Patients Treated with Systemic Therapies—Beyond *BRAF* Mutant Detection

**DOI:** 10.3390/cancers12123793

**Published:** 2020-12-16

**Authors:** Gabriela Marsavela, Peter A. Johansson, Michelle R. Pereira, Ashleigh C. McEvoy, Anna L. Reid, Cleo Robinson, Lydia Warburton, Muhammad A. Khattak, Tarek M. Meniawy, Benhur Amanuel, Michael Millward, Nicholas K. Hayward, Melanie R. Ziman, Elin S. Gray, Leslie Calapre

**Affiliations:** 1 School of Medical and Health Sciences, Edith Cowan University, Joondalup, Western Australia 6027, Australia; a.marsavela@ecu.edu.au (G.M.); m.pereira@ecu.edu.au (M.R.P.); ashleigh.mcevoy@health.wa.gov.au (A.C.M.); a.reid@ecu.edu.au (A.L.R.); lydia.warburton@health.wa.gov.au (L.W.); muhammad.khattak@health.wa.gov.au (M.A.K.); tarek.meniawy@health.wa.gov.au (T.M.M.); benhur.amanuel@health.wa.gov.au (B.A.); m.ziman@ecu.edu.au (M.R.Z.); l.calapre@ecu.edu.au (L.C.); 2 QIMR Berghofer Medical Research Institute, Herston, Brisbane, QLD 4006, Australia; peter.johansson@qimrberghofer.edu.au (P.A.J.); nick.hayward@qimr.edu.au (N.K.H.); 3 Anatomical Pathology, PathWest Laboratory Medicine, QEII Medical Centre, Nedlands, Western Australia 6009, Australia; cleo.robinson@health.wa.gov.au; 4 School of Biomedical Sciences, University of Western Australia, Crawley, Western Australia 6009, Australia; 5 Department of Medical Oncology, Sir Charles Gairdner Hospital, Nedlands, Western Australia 6010, Australia; michael.millward@uwa.edu.au; 6 School of Medicine, University of Western Australia, Crawley, Western Australia 6009, Australia; 7 Department of Medical Oncology, Fiona Stanley Hospital, Murdoch, Western Australia 6150, Australia

**Keywords:** circulating tumour DNA (ctDNA), melanoma, *BRAF*, response, targeted therapy, immunotherapy, neoantigen load, tumour mutational burden

## Abstract

**Simple Summary:**

Circulating tumour DNA (ctDNA) has been shown to be an informative biomarker in melanoma. Here we analysed plasma ctDNA in a real-world metastatic melanoma cohort. We found the kinetics of ctDNA decline are delayed in patients treated with immunotherapy compared to those receiving MAPK inhibitors. Nonetheless, decreasing ctDNA levels within 12 weeks of immunotherapy or BRAF/MEK inhibitors was strongly concordant with treatment response and significantly associated with longer progression-free survival (PFS). Furthermore, exploratory analysis of nine patients commencing anti-PD-1 therapy showed a trend of high tumour mutational burden and neoepitope load in responders compared to non-responders. The results support the use of ctDNA as a dynamic biomarker for assessment of response in melanoma patients.

**Abstract:**

In this study, we evaluated the predictive value of circulating tumour DNA (ctDNA) to inform therapeutic outcomes in metastatic melanoma patients receiving systemic therapies. We analysed 142 plasma samples from metastatic melanoma patients prior to commencement of systemic therapy: 70 were treated with BRAF/MEK inhibitors and 72 with immunotherapies. Patient-specific droplet digital polymerase chain reaction assays were designed for ctDNA detection. Plasma ctDNA was detected in 56% of patients prior to first-line anti-PD1 and/or anti-CTLA-4 treatment. The detection rate in the immunotherapy cohort was comparably lower than those with BRAF inhibitors (76%, *p* = 0.0149). Decreasing ctDNA levels within 12 weeks of treatment was strongly concordant with treatment response (Cohen’s k = 0.798, *p* < 0.001) and predictive of longer progression free survival. Notably, a slower kinetic of ctDNA decline was observed in patients treated with immunotherapy compared to those on BRAF/MEK inhibitors. Whole exome sequencing of ctDNA was also conducted in 9 patients commencing anti-PD-1 therapy to derive tumour mutational burden (TMB) and neoepitope load measurements. The results showed a trend of high TMB and neoepitope load in responders compared to non-responders. Overall, our data suggest that changes in ctDNA can serve as an early indicator of outcomes in metastatic melanoma patients treated with systemic therapies and therefore may serve as a tool to guide treatment decisions.

## 1. Introduction

In recent years, improved knowledge of melanoma pathogenesis has led to the development of BRAF and MEK inhibitors that target tumours carrying *BRAF* oncogenic mutations, accounting for 40%–50% of all melanoma cases. Similarly, antibody-mediated blockade of immune checkpoints, particularly the cytotoxic T-lymphocyte-associated antigen-4 (CTLA-4) and the programmed cell-death protein 1 (PD-1), have markedly improved patient outcome in the last 5 years [1,2,3,4,5,6]. However, a significant number of patients do not achieve sustained benefit from either targeted therapy or immunotherapy [2,3,4,5,6]. The most appropriate treatment sequence or therapy combinations that can maximise patient outcomes remains controversial [7,8]. Predictive biomarkers of therapy response that can be assessed prior to initiation of treatment and early during therapy are critical to guide clinical management of metastatic melanoma.

Analysis of tumour specific cell-free DNA (cfDNA) has been previously reported to be a reliable companion diagnostic biomarker in oncology [9,10,11,12]. In melanoma, ddP (ctDNA) is a potential non-invasive alternative to tumour tissue biopsy for molecular profiling and longitudinal disease monitoring in the metastatic setting [13,14,15,16,17,18,19,20,21,22,23]. In addition, baseline ctDNA levels and subsequent decline with treatment have been indicated as an early predictor of tumour response and clinical benefit [13,15,24,25]. To confirm the utility of ctDNA as a clinical biomarker, its ability to monitor and/or predict treatment response and clinical outcome requires further validation in a large cohort of melanoma patients, especially in those treated with immunotherapy.

In melanoma, *BRAF* mutant ctDNA has been found to be a robust biomarker for disease burden and tumour status of patients prior to and during targeted treatment [13,20,21,22]. However, many patients receiving immunotherapy, are *BRAF* wild-type (WT). Thus, the detection rate of ctDNA and the value of ctDNA-based longitudinal monitoring in non-*BRAF* melanoma patients need to be specifically assessed.

Mutations, genetic rearrangements, insertions and deletions can encode novel, cancer-specific neoantigens. Activation of T-cells is initiated by the recognition of novel peptides presented by human leukocyte antigens (HLA) complex. A high tumour mutational burden (TMB) was associated with better survival outcomes in non-small cell lung cancer (NSCLC) [26,27,28,29,30], melanoma [31,32] and other cancers. Nonetheless, the predictive value of tissue-derived TMB for immunotherapy response needs further scrutiny and standardisation [33,34,35]. In this context, ctDNA has the potential to comprehensively capture the mutational profile of all existing metastases [20]. However, whether this biomarker presents as an easily accessible and suitable tumour source for whole exome mutational load analysis and TMB measurement or neoepitope predictions in melanoma needs to be further defined.

In this study, we aimed to ascertain the clinical utility of ctDNA to inform treatment response and survival in metastatic melanoma patients receiving systemic therapy. We compared ctDNA levels, detection rates, decay kinetics and predictive value between patients treated with immune checkpoint inhibitors and targeted therapies. We also explored whether ctDNA can be used for estimating tumour mutational and neoepitope load, to predict response to immune checkpoint inhibiting therapies.

## 2. Results

### 2.1. Plasma ctDNA Detection in Melanoma Patients Commencing Systemic Therapy

**Figure 1:**
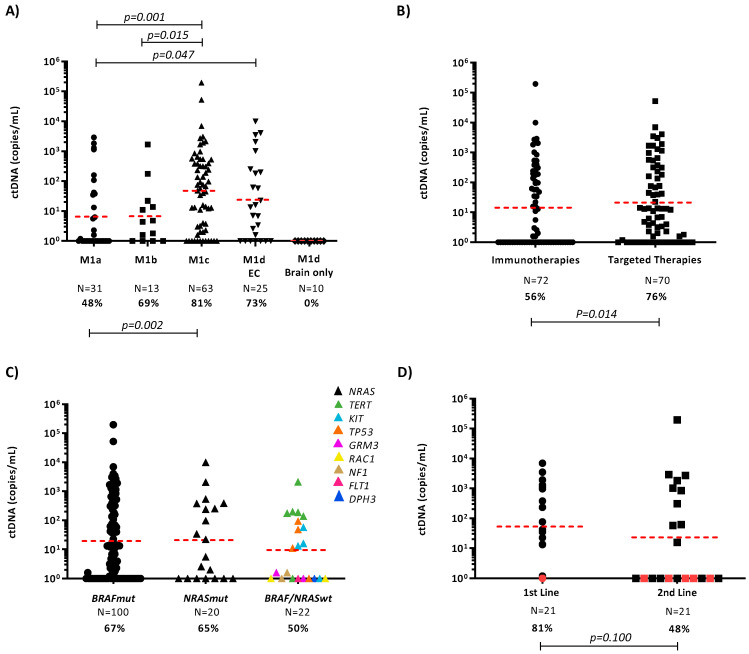
ctDNA quantification in melanoma patients prior to commencing systemic therapy. (**A**) Plasma ctDNA levels (copies/mL of plasma) in melanoma samples (N = 144), stratified by M status. M1d cases were further subdivided into those with extracranial (EC) and those with brain only metastases. Percentages denote the frequency of patients with detectable ctDNA. The geometric mean of ctDNA concentrations is indicated for each group by a dashed red line. Unpaired t-test *p*-values of the log-transformed ctDNA levels are indicated. (**B**) Dot plot diagram showing ctDNA at baseline in patients treated with immunotherapy (IT) and targeted therapy (TT). (**C**) ctDNA detection in patients with *BRAF*, *NRAS* or *BRAF/NRAS* wild-type tumours commencing first-line treatment. (**D**) ctDNA detection at first-line and second-line treatment in *BRAF* mutant patients. Red dots represent patients with intracranial disease only at the time of starting therapy.

We first evaluated the rate of ctDNA detection in 142 plasma samples collected prior to treatment initiation (Figure 1). From these 142 samples, 72 were treated with immunotherapy and 70 with targeted therapy. Both cohorts were mainly formed by middle age patients (50–69 years of age), males and with widespread disease (M1c). In the immunotherapy cohort, 30 patients were BRAF mutant (42%), 20 were NRAS mutant (28%) and 22 patients had other driver mutations that were used to track the tumour (Table S2). In this cohort, the 41 patients were treated with anti-PD1 inhibitors, 12 patients were treated with anti-CTLA-4 inhibitors and 19 patients with a combination of both (26%). On the other hand, most patients in the targeted therapy cohort were treated with combination of BRAF/MEK inhibitors, while only 5 patients were treated with single targeted therapy agent. The ctDNA detection rate was 65% overall but patients with one or more prominent visceral metastases (M1c), particularly in the liver, bone and lung, had significantly higher ctDNA detection rates when compared to those with M1a disease (*p* = 0.0015; Figure 1A).

Similarly, median ctDNA levels were significantly higher in M1c patients compared with M1a (*p* = 0.001) or M1b (*p* = 0.015). In addition, ctDNA levels in patients with M1d and extracranial disease were significantly higher compared with M1a disease (*p* = 0.047). Notably, none of the ten M1d patients with brain only metastases had detectable ctDNA (Figure 1A), indicating that ctDNA levels are influenced by the site of metastases.

### 2.2. Baseline ctDNA Detection Prior Systemic Treatments

We then compared the ctDNA detection rates in plasma collected prior to commencing treatment with immune checkpoint inhibitors or targeted agents. We observed reduced ctDNA concentrations and a significantly lower detection rate in patients receiving immunotherapy when compared to those receiving BRAFi ± MEKi (56% vs. 76%; *p* = 0.009; Figure 1B).

Due to the difference in ctDNA detection rates between the targeted therapy and immunotherapy groups, we evaluated whether the mutational target used for ctDNA analysis influenced these results. Comparison of the detection rate of ctDNA between mutational targets demonstrated no significant difference between ctDNA levels and detection rate (geometric mean: 19.2 copies/mL, 67/100, 67%) in patients with *BRAF* versus patients with other melanoma-associated mutations (geometric mean: 13.7 copies/mL, 26/42, 62%; Figure 1C).

To determine if ctDNA levels are influenced by the line of therapy, we compared ctDNA levels in 21 *BRAF* mutant patients that received first-line targeted therapy and second-line immunotherapy (Figure 1D). This sequence of treatment is commonly used for *BRAF* mutant melanoma in Australia. Despite not showing statistical significance, ctDNA detection rate was lower in patients commencing second-line treatment (81% vs. 48%, *p* = 0.100). This result is likely influenced by the effectiveness of regular radiological monitoring in identifying disease progression at low tumour burden.

### 2.3. Longitudinal ctDNA Monitoring for Prediction of Response

**Figure 2:**
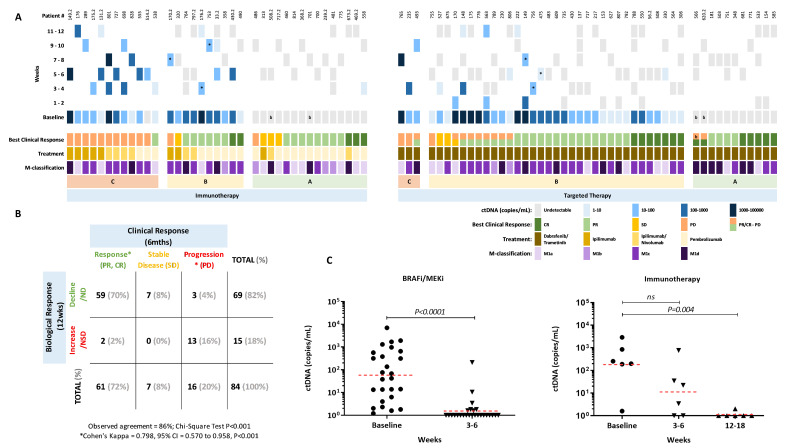
ctDNA levels early during treatment relative to clinical response. (**A**) Columns represent each patient, best clinical response, treatment type and longitudinal quantitative ctDNA results. Patients treated with immunotherapy or targeted therapy were stratified into three profile groups: A = undetectable ctDNA at baseline and during treatment with a biological response, B = detectable ctDNA at baseline that became undetectable during treatment or had a significant biological response and C = detectable/undetectable ctDNA at baseline that remained or became detectable during therapy without significant biological response. * Significant Biological Response. **^b^** Presence of only intracranial malignant disease at baseline or at PD. (**B**) Concordance between best clinical response at 6 months and biological ctDNA response within the 12 weeks of treatment. Patients categorised as clinically responders (PR/CR, N = 61), patients with stable disease (SD, N = 7) and patients with disease progression (PD, N = 16) and, ctDNA responders (Group A and B; N = 69) or non-responders (Group C; N = 15) based on their biological ctDNA response over the first 12 weeks of treatment. Abbreviations: ND=Not detectable; NSD=Non-significant decrease. (**C**) Plasma ctDNA levels at baseline and follow-up in patients that responded to targeted therapy (N = 26) and to immunotherapy (N = 6). *P*-values of paired t-tests are indicated. The geometric mean ctDNA concentration is indicated for each group by a dashed red line.

We further investigated whether ctDNA positivity at baseline and early during the treatment course were correlated with treatment response. A total of 84 patients with longitudinal blood collections within 12 weeks of treatment were included and stratified according to treatment, that is, targeted therapy (N = 47) vs. immunotherapy (N = 37) and divided into three groups depending on the ctDNA profile during the first 12 weeks of treatment (Figure 2A). Similar to that shown by Lee et al. [24] Group A consisted of patients with undetectable ctDNA levels at baseline and during 12 weeks of therapy or non-significant ctDNA changes. Group B had detectable baseline ctDNA that became undetectable or significantly reduced during treatment and group C includes patients that were either ctDNA positive or negative at baseline with static or significantly increased levels during the first 12 weeks of therapy. Overall, groups A and B represented patients that showed a biological response, evidenced by undetectable or a significant reduction in ctDNA levels and group C was comprised of patients that did not show a biological response, that is, detectable or non-significant reduction in ctDNA levels.

An 86% observed agreement was found between the best clinical response within 6 months from treatment initiation and the biological response offered by longitudinal ctDNA monitoring (72/84). Notably, a subset of seven patients without objective response or unequivocal disease progression, who were treated with either immunotherapies (N = 3) or targeted therapies (N = 4), had a biological response. A strong agreement was found between the biological and the clinical response (Figure 2B; κ = 0.798, 95% CI 0.570 to 0.958, N = 77, *p* < 0.001), when these seven patients were excluded from the analysis. Discordance was observed in five patients (5/77, 6%), with three patients noted to have no detectable or significant decrease in ctDNA levels despite having clinical progression (PD) in subcutaneous lesions (Patient #170.2 and 755), lymph nodes (755), muscle (755 and 486) and brain (170.2). The PD lesions observed in patient 170.2 were in the subcutaneous tissue and brain. By contrast, two patients (538 and 493) were found to have a clinical response to pembrolizumab and dabrafenib/trametinib, respectively but no biological response was observed.

We next compared the biological ctDNA response with longitudinal blood collection for a period of 24 weeks after starting treatment. In this cohort, most patients treated with anti-CTLA-4 and anti-CTLA-4/PD-1 did not show radiological response to therapy and their ctDNA levels remained high (Figure S2A,B). By contrast, 17 of the 21 (81%) patients receiving anti-PD-1 immunotherapy had a partial response (PR) or complete response (CR) (Figure S2C). The clinical response rate in the targeted therapy cohort was also high (41/47, 87%) but a number of these patients (10/41, 24%) developed resistance and relapsed within the first 24 weeks of therapy, with 9 of them demonstrating rebounding ctDNA levels (Figure S2D).

We analysed patients with objective clinical response that had detectable ctDNA at baseline and assessable follow-up samples. Within these groups, ctDNA dropped significantly by 3-6 weeks in the targeted therapy cohort (N = 26, *p* < 0.0001; Figure 2C). In contrast, most patients (67%) who responded to immunotherapy had detectable ctDNA levels at first follow-up and, only had the significant drop to undetectable levels on their second follow-up by 12-18 weeks (*p* = 0.004, Figure 2C).

### 2.4. Longitudinal ctDNA Monitoring for Prediction of Survival

We evaluated whether the ctDNA changes during the first 12 weeks of treatment (groups A, B or C) had prognostic value in patients treated with immunotherapy. For the survival analysis, patients receiving single-agent immunotherapy ipilimumab (N = 8) were excluded due to their poor response rate and rapid transition into anti-PD-1, which may confound survival analysis. Clinical characteristics across the three groups were similar for age, sex, tumour stage, the prevalence of brain metastases and prior lines of treatment (Table S3).

**Figure 3:**
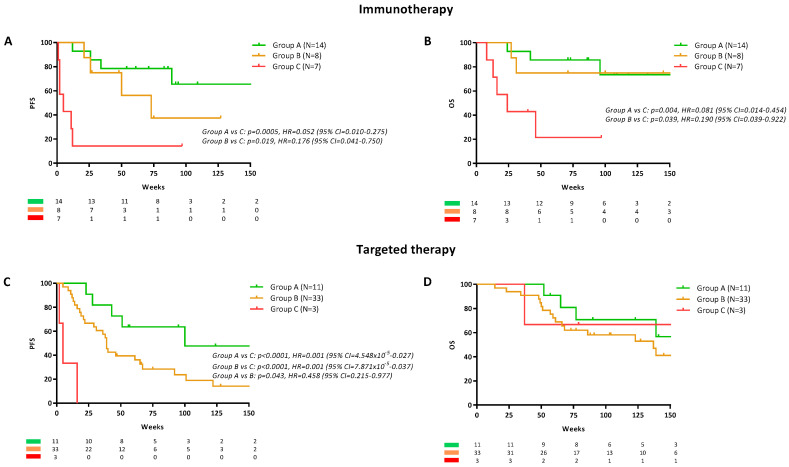
ctDNA levels early during treatment relative to survival. Progression free survival (PFS) and overall survival (OS) curves for patients treated with (**A**,**B**) immunotherapy or targeted therapy (**C**,**D**) stratified into the three previously detailed profile groups A, B and C. Cox regression *p*-values, Hazard Ratio (HR) and 95% confidence intervals (CI) are indicated for each plot.

In patients receiving immunotherapy, groups A and B had significantly longer progression free survival (PFS) and overall survival (OS) compared to group C (Figure 3A,B). Median PFS for groups B and C was 73 and 5 weeks respectively but was not reached for group A. The hazard ratio (HR) was 0.052 (95% CI = 0.010 to 0.275, *p* = 0.0005) for group A and 0.176 (95% CI = 0.041 to 0.750, *p* = 0.019) for group B when compared with group C. There was no statistical difference in the PFS of groups A and B (*p* > 0.05). Median OS for group B and C were 150 and 24 weeks respectively but was not reached for group A (Figure 3B). The HR was 0.081 (95% CI = 0.014 to 0.454, *p* = 0.004) for group A and 0.190 (95% CI = 0.395 to 0.922, *p* = 0.039) for group B when compared with group C. There was no statistical difference in the OS of groups A and B (HR = 0.705, 95% CI = 0.134 to 3.693, *p*>0.05). In a multivariable Cox regression model, ctDNA kinetics in group C was found to be an independent predictor of shorter PFS (HR = 15.11, 95% CI = 3.33 to 68.54, *p* < 0.001) and OS (HR = 16.01, 95% CI = 2.44 to 105.07, *p* = 0.004; Table S4). The model also indicated that age over 65 years and M1c/d stage were independent predictors of short OS.

Notwithstanding the low number of samples in Group C (N = 3), in patients treated with targeted therapy (Figure 3C), group A had longer PFS when compared to group B and C. Median PFS was 100, 39 and 5 weeks for group A, B and C, respectively. When compared with group A, the HR was 0.458 (95% CI = 0.215 to 0.977, *p* = 0.043) for group B and 0.001 (95% CI = 4.548 × 10^−5^ to 0.027, *p* < 0.0001) for group C. Despite the differences in the PFS between these groups, there was no difference between their median OS (Figure 3D, *p* > 0.05). A multivariable Cox regression model, found that ctDNA kinetics in group B is an independent predictor of decreased PFS (HR = 3.45, 95% CI = 1.02 to 11.65, *p* = 0.046) but not of OS (*p* > 0.05; Table S4). The presence of brain metastases only and an Eastern Cooperative Oncology Group (ECOG) performance status above 3 were also predictors of shorter PFS and OS. Due to the low number of samples in group C (N = 3), these patients were excluded from the analysis.

### 2.5. Measuring Mutational Burden Using ctDNA

Plasma ctDNA analysis constitutes an attractive approach for real-time assessment of tumour mutational profile and alleviates caveats associated with tissue biopsies including tumour heterogeneity. Here, we determined the feasibility of quantifying mutational load in patient-derived cfDNA. We screened melanoma patients treated with the anti-PD-1 inhibitor as a first- or second-line treatment, with a ctDNA fraction of more than 7% abundance by ddPCR. Nine patients were selected and dichotomised according to their best clinical response to therapy, with responders noted as having either a PR, CR or prolonged stable disease (SD). Non-responders are those without clinical or objective response and who had progressive disease (PD) within 6 months of treatment initiation. Clinical characteristics for these patients are described in Table S5.

**Figure 4:**
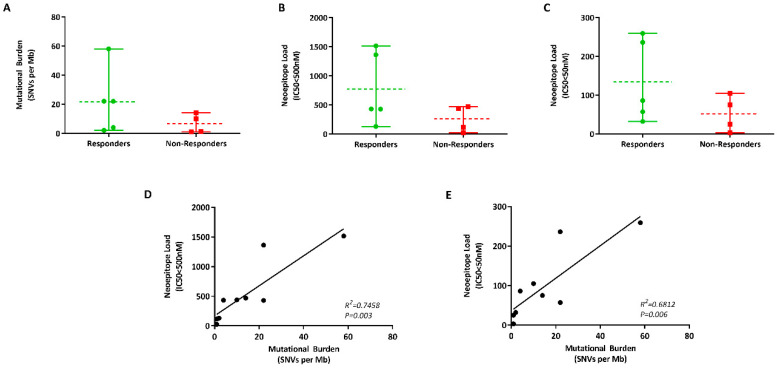
ctDNA as a tumour source for mutational burden analysis. (**A**) Vertical scatter plot of the difference in the mutational burden (number of single-nucleotide variants (SNVs per Mb of DNA) in responders (green) and non-responders (red) to anti-PD-1 blockade. Graphs indicating the difference in the number of low—IC50 < 500 nM (**B**) and high—IC50 < 50 nM (**C**) affinity neoantigens in melanoma patients treated with immunotherapy. Correlation between the mutational burden and neoepitope loads at IC50 < 500 nM (**D**) and IC50 < 50 nM (**E**).

Mutational data were obtained from the nine patients, with the number of mutations ranging from 1-58 per Mb of DNA (Table 1). While patients that responded to anti-PD-1 inhibitor had higher TMB compared to non-responders (mean: 21 vs. 6 per Mb), the difference was not statistically significant (Figure 4A). Nonetheless, our results may be confounded by the small sample size analysed for TMB.

As mutational burden alone did not explain clinical benefit from anti-PD-1 inhibitors, we hypothesised that the presence of specific tumour neoantigens might explain the varied dichotomised patients that are likely to benefit from this immunotherapy. To identify these neoepitopes, the HLA-I phenotype of each patient were identified and the bioinformatics pipeline for pVACSeq (https://github.com/griffithlab/pVAC-Seq) was used for neoepitope prediction.

The number of predicted neoepitopes with a binding affinity of IC50 < 500 nM ranged from 25-1516 and was higher in responders (mean = 774) versus non-responders (mean = 262) to immunotherapy (Figure 4B). The number of predicted neoepitopes with a strong binding affinity (IC50 < 50nM) ranged from 3–259 and was similarly higher in responders compared to non-responders (mean = 134 vs. 52, Figure 4C). However, the difference in the number of neoepitopes in these two groups was again not significant (*p* > 0.05). Nonetheless, the number of neoepitopes correlated with the mutational burden (Figure 4D,E). Overall, there was a trend that high neoepitope load was associated with response to anti-PD-1 treatments. Nevertheless, three of the five responders had neoepitope loads in the same range as the non-responders, indicating that at a singular patient level, this parameter alone cannot be used for treatment decisions.

## 3. Discussion

The prognostic value of ctDNA in melanoma patients has been previously shown by number of studies [17,18,20,24,25]. In this study, we found ctDNA detectability at baseline and during treatment course to be a strong predictor of clinical outcome. In particular, we showed that high levels of ctDNA at baseline and throughout the first 12 weeks of treatment were indicative of poor survival outcome in melanoma patients receiving first-line immune checkpoint inhibitors as well as on those receiving targeted therapies. Moreover, patients with undetectable ctDNA at baseline, who remained ctDNA negative during treatment, have a longer time to progression irrespective of treatment. Notably, detectability of ctDNA and its resolution during treatment was also associated with good clinical outcome in patients treated with immunotherapy and targeted therapies. In addition, we describe for the first time a different ctDNA pattern of response in targeted therapy and in immunotherapy.

Overall, our findings underscore the suitability of ctDNA as a prognostic biomarker for the currently available treatments of melanoma patients. Our findings indicate that ctDNA is most informative as an early indicator of clinical response. In fact, we found a significant concordance between baseline ctDNA levels and response to first-line immunotherapy and targeted therapy. The decline in ctDNA levels was found to be highly concordant with the radiological response to treatment, while increasing ctDNA levels was correlated with disease progression. These results are supported by previous findings [24,36] and further demonstrated the ability of ctDNA to accurately reflect the disease status of patients, making it a valuable surrogate or companion biomarker for patient surveillance during treatment.

Interestingly, we found a low response rate amidst patients treated with anti-PD-1 plus anti-CTLA-4, in contrast with that observed in clinical trials [3,37]. The patients in our combined immunotherapy cohort had extensive brain metastases and/or widespread disease, which may have reduced the response rates. Moreover, very few patients in our cohort were treated with combined immunotherapy and therefore the response rates observed here may not necessarily reflect that of previous studies.

We also want to highlight the difference in the rate of ctDNA decay between patients treated with targeted therapy and immunotherapy. In this study, we observed a delayed velocity of ctDNA decay in patients that respond to immunotherapy compared to patients undergoing targeted therapy. This data reflects the time interval necessary to unleash an immune response to cancer [38], which needs to be taken as an important consideration when monitoring response to different types of treatment through a liquid biopsy. The current treatment approach for melanoma is based on evaluating disease progression, followed by treatment modification to potentially improve patient outcomes and discontinue ineffective therapy. Our data suggest that an observation period may be required prior to conclusive evaluation of therapeutic benefits to immunotherapy and treatment modification decisions.

While ctDNA was found to be a reliable prognostic and surveillance biomarker, it is not without limitations. A significant roadblock for ctDNA analysis in this study was the low detection rate of ctDNA prior to anti-PD-1 and/or anti-CTLA-4 treatment compared with targeted therapy. As indicated above, most patients with detectable ctDNA have prominent visceral metastases, particularly to the liver. The variation of tumour cell turnover at different metastatic sites may have an impact on the detectability of ctDNA. In addition, the low detection rate may have been affected by the specificity of the assay used for ctDNA analysis. Aside from our in-house *BRAF* assays, which have been previously reported to have high specificity and sensitivity [39], assays for other mutations have a lower limit of detection due to noise [21]. Differences in assay threshold may also affect the detection rate of ctDNA in melanoma patients treated with immune-checkpoint inhibitors. Thus, the site of metastases and the assay specificity of the mutational target for ctDNA analysis appears to highly influence the variation in the detection rate observed in this study.

Previous studies have demonstrated the predictive value of tissue-derived mutational and neoepitope load for immunotherapy response in NSCLC [26,27] and melanoma [31]. In this study, we also explored the potential utility of ctDNA for mutational and neoepitope load analysis in melanoma. Gandara et al. [30] demonstrated the utility of blood tumour mutational burden as a clinically-actionable biomarker for anti-PD-L1 in NSCLC. Similarly, our exploratory analysis also demonstrated that whole exome sequence (WES)-defined molecular analysis for clarifying tumour mutational burden in ctDNA is possible. In our cohort, mutational load was unable to discriminate between responders and non-responders to anti-PD-1 inhibitor. Nonetheless, we observed a trend showing high neoepitope load in patients that achieved clinical benefit to anti-PD-1 blockade. The small sample size was not sufficient to discriminate between responders and non-responders to immunotherapy. These findings may be confounded by the small sample size mostly consisting of patients with high levels of ctDNA (>7% frequency abundance). WES analysis imposed the need to select for patients with high ctDNA fraction, which excluded most samples in our cohort. On the other hand, mutational burden derived from targeted sequencing has been previously shown to be sufficient for stratifying responders and non-responders to immunotherapy [30]. Thus, a targeted approach, with the addition of unique molecular identifiers (UMI), may be more fitting for ctDNA mutational burden analysis, as it will be able to control for PCR errors and allow interrogation of variants at low allelic fraction (<1%).

## 4. Materials and Methods

### 4.1. Patients

We analysed a total of 142 plasma samples collected prior to commencing systemic therapy and 227 follow-up samples collected within 24 weeks of treatment initiation from 118 metastatic melanoma patients enrolled in the study between 2013–2018 at Sir Charles Gairdner Hospital (SCGH) and Fiona Stanley Hospital (FSH) in Perth, Western Australia. A subset of 24 patients were considered as baseline for their first- and second-line therapy. Additional details of study design and patient inclusion or exclusion criteria in the different analyses can be found in Figure S1. The study was conducted in accordance with the Declaration of Helsinki and the protocol was approved by the Human Research Ethics Committee from Edith Cowan University (No. 11543 and No. 18957) and Sir Charles Gairdner Hospital (No. 2013-246). Written consent was obtained from all patients under approved human research ethics committee which complied with the Declaration of Helsinki. Patients were clinically monitored with median follow-up duration of 113 weeks (range: 28–286 weeks). Patient characteristics and clinical parameters are summarised in Table S1.

### 4.2. Treatment Response and Disease Progression Assessment

Tumour disease responses were assessed radiologically by computed tomography (CT) and/or ^18^F-labeled fluorodeoxyglucose positron emission tomography (FDG-PET) scans at two to three monthly intervals. Patients were defined as responders if they had significant reduction in tumour size by the RECIST 1.1 on CT or FDG-PET scan as per the treating clinician or presented a durable stable disease lasting more than 6 months. PFS was defined as the time interval between the start of therapy and the date of first clinical progression. OS was defined as the time interval between the start of therapy and death. Additionally, metastatic melanoma patients were stratified into four M-subcategories at baseline based on the location of the metastases [40].

### 4.3. Statistics

Differences between ctDNA levels were estimated by unpaired t-test from the log transformed data. Paired t-test was used to evaluate the difference between ctDNA levels at first and second-line treatment in *BRAF* mutant patients. Differences between the detection rates were assessed using one-sided Fisher’s exact test. PFS and OS were estimated using the Kaplan-Meier method and differences were evaluated using Mantel-Cox tests. Concordance between the clinical response and the ctDNA kinetics was calculated using Fisher’s exact test and the Cohen kappa measure with 95% CI from 1000 bias-adjusted and accelerated bootstrap (BCa) replications. Statistical difference between baseline and follow-up ctDNA levels from the same individuals were assessed by the Poisson test, using the minimum and maximum values plus total droplet counts as analytical variables [41]. Frequencies and percentages by each group along with their corresponding *P*-values of two-sided chi-squared or the Fisher’s exact test are reported in Table S3. All covariates were entered into a Cox proportional hazard model for multivariable analysis. The final model was chosen using a backward conditional selection procedure for selection of predictors of PFS and OS as reported in Table S4. The unpaired two-tailed t-test was used to compare mutational and neoepitope load between patients that were responders or non-responders to anti-PD-1 immunotherapy. Pearson correlation was used to determine the correlation between mutational burden and neoepitope load. All statistical analyses were performed using R version 5.2 (https://www.r-project.org/), GraphPad Prism version 5 (GraphPad Software, Inc., San Diego, CA, USA) and SPSSv22.0 (IBM, Armonk, NY, USA). Results were considered statistically significant at *p* < 0.05.

## 5. Conclusions

In conclusion, ctDNA has significant clinical value as a biomarker of prognosis and therapeutic response for melanoma. Nonetheless, limitations inherent to ctDNA analysis need to be clearly defined and thoroughly addressed prior to its implementation in the clinic.

## Figures and Tables

**Table 1:** Mutational burden and predicted neoepitope load of nine melanoma patients.

Response	Sample ID	Mutational Burden (per Mb)	Predicted Neoantigens IC50 < 500 nM	Predicted Neoantigens IC50 < 50 nM
Responders (*R*)	MP0104	22	1362	236
	MP0105	2	131	32
	MP0201	4	432	86
	MP0302	58	1516	259
	MP0303	22	429	57
Non- Responders (*NR*)	MP0102	10	439	105
	MP0103	1	114	25
	MP0301	1	25	3
	MP0304	14	469	75

## Supplementary Materials

The following are available online at https://www.mdpi.com/2072-6694/12/12/3793/s1, Additional Material and Methods. Figure S1: Flow chart showing group of samples included in the analyses, Figure S2: Kinetics of ctDNA decay, Table S1: Demographic, clinicopathologic and treatment characteristics of included samples, Table S2: Specificity of ddPCR assays, Table S3: Clinical characteristics at baseline of the melanoma patients categorised in Groups A, B and C included in the survival analysis (N = 76), Table S4: Univariable and multivariable Cox proportional-hazards regression analysis for associations between ctDNA levels and survival, Table S5: Clinical characteristics of melanoma patients in this pilot cohort.
