# Supplementary material for: The Prognostic Impact of Circulating Tumour DNA in Melanoma Patients Treated with Systemic Therapies—Beyond BRAF Mutant Detection
Source: Cancers (Basel). 2020 Dec 16;12(12):3793. doi: 10.3390/cancers12123793 (PMC7765660; doi:10.3390/cancers12123793)
Supplement: Supplementary file 1 [file cancers-12-03793-s001.pdf]

cancers

Supplementary Materials

The Prognostic Impact of Circulating Tumour DNA in Melanoma Patients Treated with Systemic Therapies—Beyond *BRAF* Mutant Detection

Gabriela Marsavela [1], Peter A. Johansson [2], Michelle R. Pereira [1], Ashleigh C. McEvoy [1], Anna L. Reid [1], Cleo Robinson [3,4], Lydia Warburton [1,5], Muhammad A. Khattak [1,6,7], Tarek M. Meniawy [1,5,6], Benhur Amanuel [1,3,6], Michael Millward [5,6], Nicholas K. Hayward [2], Melanie R. Ziman [1,4], Elin S. Gray [1,*] and Leslie Calapre [1]

Additional Materials and Methods

Plasma Samples Preparation and cfDNA Extractions

Blood samples were collected prior to initiation of treatment and during subsequent follow-ups. For most patients, bloods were collected within a week prior treatment initiation (102/142, 72%), extending for some patients to up to -51 days, with no treatment received during this period. Blood was collected into EDTA vacutainer or Cell-Free DNA BCT® (Streck, La Vista, NE) tubes. Within 24 hours of blood collection, plasma was separated by centrifugation at 300 g for 20 minutes, followed by a second centrifugation at 4700 g for 10 minutes. All isolated plasma was stored at -80°C until extraction. Plasma cell-free DNA (cfDNA) was isolated from 1–5 mL of plasma using QIAamp Circulating Nucleic Acid Kit (Qiagen, Hilden, Germany) as per the manufacturer's instructions. The recovered cfDNA was eluted in 40 µL AVE buffer (Qiagen, Hilden, Germany) and stored at -80°C until ctDNA quantification by droplet digital polymerase chain reaction (ddPCR).

Tissue Analysis

Mutational profile of *BRAF* WT patients were identified from tissue biopsies as previously described by Calapre et al. [1]. A custom targeted next generation sequencing panel of 30 melanoma-associated genes (Illumina, San Diego, CA, USA) with 950 amplicons and an Illumina MiSeq instrument were used to identify mutational targets for circulating tumour DNA (ctDNA) analysis in *BRAF* WT patients. Genomic variants were annotated using the Illumina Variant Studio 3.0 software (Illumina). Mutational targets were selected based on the criteria previously described [1].

Plasma ctDNA Analysis

Commercially available and/or customised probes were used to analyse ctDNA by ddPCR. Droplets were generated using an Automatic Droplet generator QX200 AutoDG (Bio-Rad, Hercules, CA). Amplifications were performed in 40 µL reactions using cycling conditions previously described [2]. Twenty-six different mutation variants in 10 different genes were utilised. Customised primers and probes for *TERT* and *DPH3* promoter mutation analyses were performed as previously reported by McEvoy et al. [3] and Calapre et al. [1], respectively. Limit of blank for the ctDNA assays was determined using normal plasma samples from at least 10 healthy controls. Levels of ctDNA were defined based on the level of false positive droplets as previously specified in Calapre *et al.* [1] or are detailed in Table S2. Samples yielding copies/mL of plasma equal or below the maximum false positive concentration were deemed ctDNA negative.

Whole Exome Sequencing

The concentrations of cfDNA used for WES ranged from 1 to 7 ng/uL of cfDNA, with ctDNA fraction >7% abundance. Whole exome sequencing (WES) was carried out using the Exome-seq

Agilent V6 capture Kit (Agilent) by Novogene (Hong Kong, China). Sequence reads were aligned against human reference genome (hg19) using the Burrows-Wheeler aligner (BWA). Duplicate reads were marked with Picard Tools, reads were realigned against known indels and base qualities recalibrated using Genome Analysis Toolkit. As BWA assumes a unimodal distribution of fragment size, we adjusted the Proper Pair bit in read pairs following the approach in BWA but the fragment sizes were fitted against a mixture of two Gaussian models.

Somatic variants were identified with an in-house tool using the statistical framework described by Li et al. [4]. We used a model assuming diploid germline and calculated the phred-scaled likelihoods for possible genotypes. The tumour sample was modelled as a mixture of tumour and normal cell DNA and likelihoods were calculated for an array of different variant allele frequencies. The constrained log-likelihood ratio (CLR) was calculated and variants with CLR score of < 70 were excluded from further analysis. Identified variants were further annotated using ANNOVAR.

Neoepitope Load Prediction

To predict neoantigens formed by the somatic variants, we used pVACseq v4.0.9 [5] with epitope lengths 8-11 and NetMHCpan binding predictions [6]

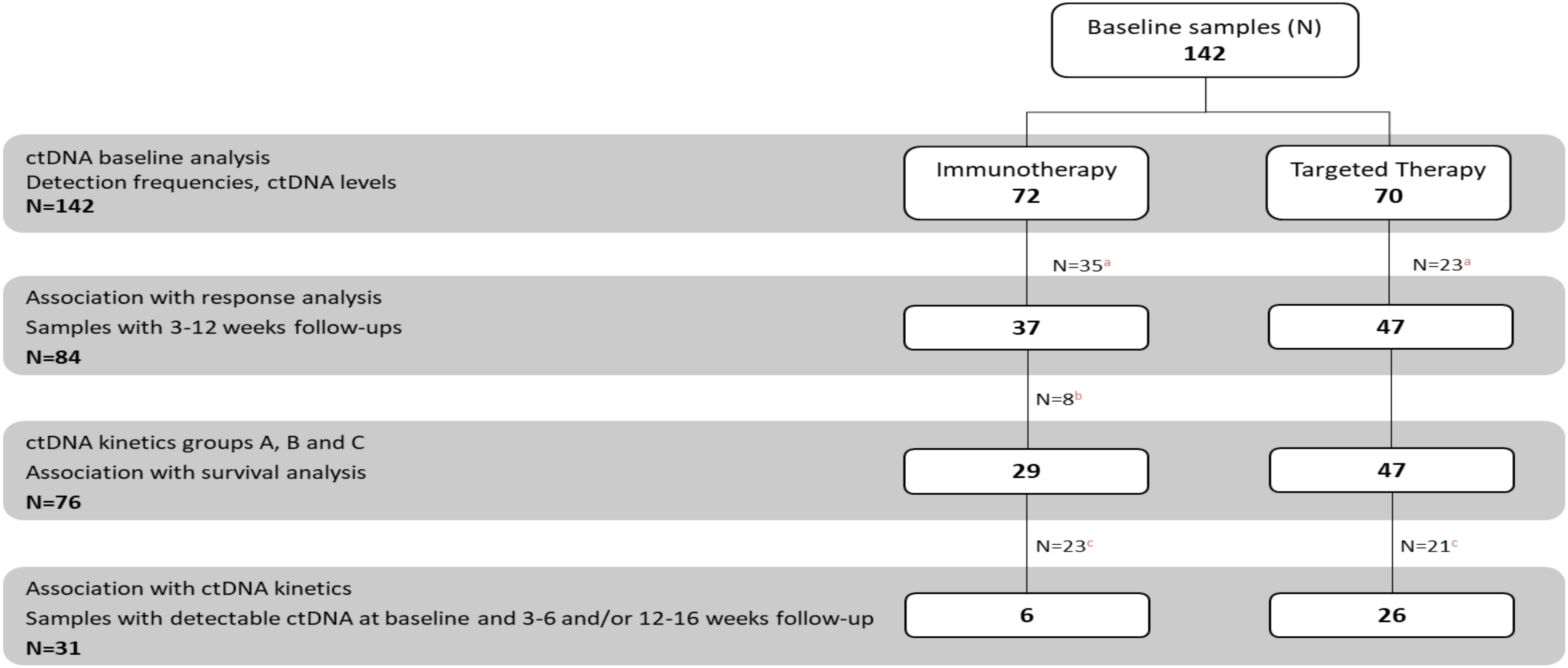

[a] Exclusion of samples without follow-up within 12 weeks of therapy. [b] Exclusion of ipilimumab cases. [c] Exclusion of samples without detectable ctDNA at baseline and assessable 3-6 and/or 12-16 week follow-ups.

Figure S1. Flow chart showing group of samples included in the analyses.

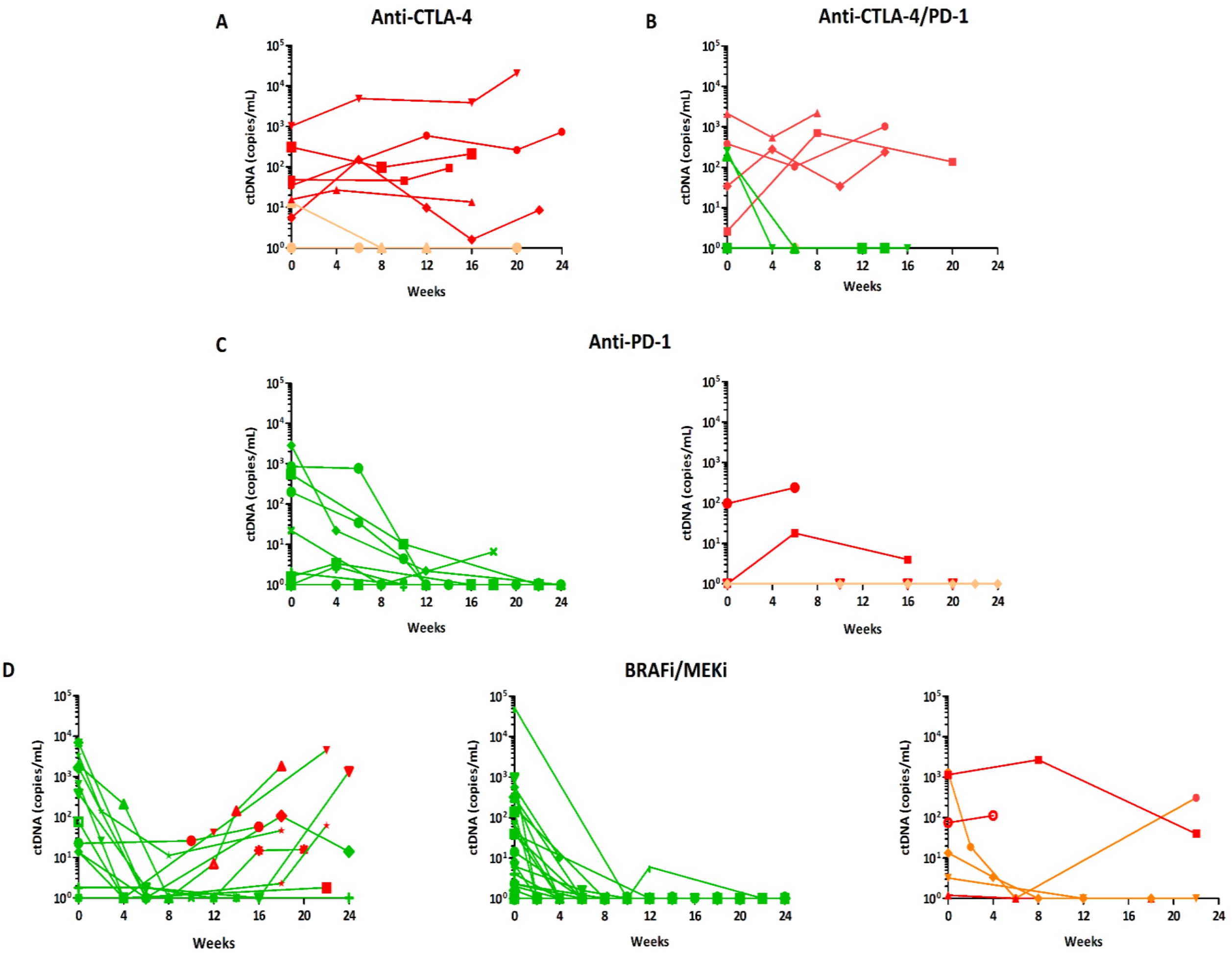

Figure S2. Kinetics of ctDNA decay. Time course of biological response for patients undergoing first or second-line treatment with (**A**) anti-CTLA-4 (N = 8), (**B**) anti-CTLA-4 plus anti-PD-1 (N = 8), (**C**) anti-PD-1 (N = 21) or (**D**) targeted therapy (BRAF/MEKi) (N = 47). Solid lines in green, orange and red denotes treatment responders, stable disease and non-responders, respectively. Solid lines in green with red symbol represents patients that developed resistance to targeted therapy.

Table S1. Demographic, clinicopathologic and treatment characteristics of included samples.

**Variable**	**All samples N = 142**	**Immunotherapy N = 72 (%)**	**Targeted Therapy N = 70 (%)**
**Age**			
	20-49	11 (15)	20 (29)
	50-69	38 (53)	31 (44)
	70-99	23 (32)	19 (27)
**Sex**			
	Female	18 (25)	25 (36)
	Male	54 (75)	45 (64)
**M Classification**			
	M1a	16 (22)	15 (21)
	M1b	7 (10)	6 (9)
	M1c	31 (43)	32 (46)
	M1d	18 (25)	17 (24)
**Mutational Status**			
	BRAF Mutant	30 (42)	70 (100)
	NRAS Mutant	20 (28)	
	Others	22 (30)	
**Treatment**			
	**Anti-PD1 inhibitor**		
	Pembrolizumab	40 (56)	
	Nivolumab	1 (1)	
	**Anti-CTLA-4 inhibitor**		
	Ipilimumab	12 (17)	
	**Anti-PD-1 plus anti-CTLA-4 inhibitor**		
	Ipilimumab/Nivolumab	19 (26)	
	**BRAFi**		
	Vemurafenib		4 (6)
	Dabrafenib		1 (1.5)
	**BRAFi plus MEKi**		
	Dabrafenib/Trametinib		64 (91)
	Vemurafenib/Cobimetinib		1 (1.5)

Table S2. Specificity of droplet digital polymerase chain reaction (ddPCR) assays.

Assay	Healthy controls		Maximum false positive concentration (copies/mL)
	Positive	Negative	
BRAF V600E	0	22	0
BRAF V600K	0	23	0
BRAF V600R	0	24	0
BRAF V600E2	1	12	1
BRAF K601E	3	7	2
BRAF L597Q	0	16	0
NRAS Q61K	0	19	0
NRAS Q61L	3	9	7
NRAS Q61P	1	9	2
NRAS Q61R	7	24	9
NRAS G12D	4	6	3
NRAS G13D	4	6	5
TERT C228T	*1*	29	*2*
TERT C250T	2	18	4
DPH3 C8T	1	10	2
FLT1 T543I	1	17	1
FLT1 E011K	3	7	9
NF1 P1851S	3	11	3
KIT L576P	1	11	2
KIT V559A	0	12	0
KIT W557R	0	13	0
RAC1 P29S	0	12	0
TP53 R248Q	6	8	2
TP53 R248W	2	14	2
TP53 R158H	1	9	2
GRM3 E538K	0	10	0
GRM3 S491L	6	12	6

Table S3. Clinical characteristics at baseline of the melanoma patients categorised in Groups A, B and C included in the survival analysis (N = 76).

	Immunotherapy					Targeted Therapy				
	Group A	Group B	Group C	Total	*P*-value* (A/B/C)	Group A	Group B	Group C	Total	*P*-value* (A vs B)
**Variable**	N=14 (%)	N=8 (%)	N=7 (%)	N=29 (%)		N=11 (%)	N=33 (%)	N=3 (%)	N=47 (%)	
**Age**										
≤65	5 (36)	6 (75)	3 (43)	14 (48)	0.196	5 (45)	18 (55)	2 (67)	25 (53)	0.601
>65	9 (64)	2 (25)	4 (57)	15 (52)		6 (55)	15 (45)	1 (33)	22 (47)	
**Gender**										
Female	3 (21)	1 (12)	4 (57)	8 (28)	0.120	5 (45)	10 (30)	1 (33)	16 (34)	0.359
Male	11 (79)	7 (88)	3 (43)	21 (72)		6 (55)	23 (70)	2 (67)	37 (66)	
**M Classification**										
M1a/M1b	9 (67)	2 (25)	1 (14)	12 (41)	**0.049**	2 (18)	9 (27)	1 (33)	12 (26)	0.546
M1c/M1d	5 (36)	6 (75)	6 (86)	17 (59)		9 (82)	24 (73)	2 (67)	35 (74)	
**Brain metastasis**										
Yes	3 (21)	2 (25)	1 (14)	6 (21)	0.873	6 (55)	5 (15)	1 (33)	12 (26)	**0.009**
No	11 (79)	6 (75)	6 (86)	23 (79)		5 (45)	28 (85)	2 (67)	36 (74)	
**Brain only metastasis**										
Yes	2 (14)			2 (7)	-	2 (18)			2 (4)	**0.012**
No	12 (86)	8 (100)	7 (100)	27 (93)		9 (82)	33 (100)	3 (100)	45 (96)	
**ECOG status**										
0	10 (71)	4 (50)	3 (43)	17 (59)	0.385	9 (82)	21 (64)	2 (67)	32 (68)	0.262
1-3	4 (29)	4 (50)	4 (57)	12 (41)		2 (18)	12 (36)	1 (33)	15 (32)	
**LDH levels**										
Normal	7 (50)	2 (25)	1 (14)	10 (34)	-	8 (73)	13 (39)		21 (44)	-
Abnormal	1 (7)	4 (50)	3 (43)	8 (28)			13 (39)	1 (33)	14 (30)	
Not available	6 (43)	2 (25)	3 (43)	11 (38)		3 (27)	7 (22)	2 (67)	12 (26)	
**Prior lines of therapy**										
Yes	6 (43)	4 (50)	1 (14)	11 (38)	0.316	1 (6)	2 (6)		3 (6)	0.729
No	8 (57)	4 (50)	6 (86)	18 (62)		10 (94)	31 (94)	3 (100)	44 (94)	
**BRAF mutational status**										
BRAF Mutant	6 (43)	2 (25)	1 (14)	9 (31)	0.373	11 (100)	33 (100)	3 (100)	47 (100)	-
BRAF WT	8 (57)	6 (75)	6 (86)	20 (69)						

*The *P*-value was calculated using the Chi-square test

Table S4. Univariate and multivariate Cox proportional-hazards regression analysis for associations between ctDNA levels and survival.

	**Group A/B/C Immunotherapy**				**Group A/B Targeted Therapy**			
**UNIVARIATE**								
**Variables**	**Progression free survival**		**Overall survival**		**Progression free survival**		**Overall survival**	
	HR (95% CI)	*P*-value	HR (95% CI)	*P*-value	HR (95% CI)	*P*-value	HR (95% CI)	*P*-value
Age (≤65 vs. >65)	0.46 (0.15 - 1.40)	0.174	0.83 (0.25 - 2.73)	0.761	1.18 (0.60 - 2.34)	0.625	1.06 (0.45 - 2.50)	0.894
Gender (female vs. male)	0.58 (0.19 - 1.75)	0.333	1.24 (0.31 - 5.03)	0.758	1.49 (0.71 - 3.12)	0.294	1.17 (0.47 - 2.90)	0.738
M Classification (M1a/b vs. M1c/d)	1.93 (0.64 - 5.83)	0.242	3.81 (0.80 - 18.08)	0.092	0.97 (0.44 - 2.15)	0.947	1.54 (0.52 - 4.60)	0.436
Brain metastasis (no vs. yes)	1.98 (0.60 - 6.50)	0.262	1.795 (0.46 - 7.01)	0.404	1.57 (0.74 - 3.34)	0.240	1.765 (0.71 - 4.39)	0.223
Brain only metastasis (no vs. yes)	-	-	-	-	2.76 (0.63 - 12.18)	0.179	4.19 (0.92 - 19.00)	0.063
ECOG (0 vs. 1-3)	1.52 (0.53 - 4.34)	0.437	1.82 (0.55 - 6.01)	0.323	**2.34 (1.16 – 4.75)**	**0.018**	**4.56 (1.88 – 11.06)**	**0.001**
Prior lines of therapy (no vs. yes)	1.00 (0.35 - 2.89)	0.998	0.63 (0.18 - 2.20)	0.464	1.12 (0.34 - 3.67)	0.852	0.57 (0.07 - 4.24)	0.580
BRAF mutational status (WT vs. mut)	0.77 (0.35 - 2.31)	0.636	0.72 (0.20 - 2.53)	0.607	-	-	-	-
ctDNA levels								
Group A vs Group B	2.07 (0.51 - 8.36)	0.305	1.61 (0.32 - 8.06)	0.563	2.590 (0.99 - 6.77)	0.052	1.73 (0.57 - 5.18)	0.329
Group A vs Group C	**9.16 (2.47 - 33.93)**	**0.001**	**7.46 (1.70 - 32.76)**	**0.008**				
**MULTIVARIATE**								
**Variables**	**Progression free survival**		**Overall survival**		**Progression free survival**		**Overall survival**	
	HR (95% CI)	*P*-value	HR (95% CI)	*P*-value	HR (95% CI)	P-value	HR (95% CI)	*P*-value
Age (≤65 vs. >65)	0.32 (0.09 - 1.15)	0.081	**7.33 (1.01 - 53.25)**	**0.049**				
Gender (female vs. male)								
M Classification (M1a/b vs. M1c/d)			**7.75 (1.02 - 58.76)**	**0.048**				
Brain metastasis (no vs. yes)	3.39 (0.92 - 12.54)	0.068						
Brain only metastasis (no vs. yes)					**13.27 (2.02 - 87.23)**	**0.007**	**8.77 (1.74 - 44.31)**	**0.009**
ECOG (0 vs. 1-3)					**2.28 (1.09 - 4.76)**	**0.029**	**5.69 (2.23 - 14.51)**	**0.000**
Prior lines of therapy (no vs. yes)								
BRAF mutational status (WT vs. mut)								
ctDNA levels								
Group A vs Group B	1.57 (0.37 - 6.61)	0.539	0.69 (0.12 - 3.86)	0.671	**3.45 (1.02 – 11.65)**	**0.046**		
Group A vs Group C	**15.11 (3.33 - 68.54)**	**0.000**	**16.01 (2.44 - 105.07)**	**0.004**				

Table S5. Clinical characteristics of melanoma patients in this pilot cohort.

Sample ID	BRAF Status	Treatment	Best Clinical Response	Previous Immunotherapy	Start of Treatment	Treatment Completion or Latest Clinic	Length of Treatment (wks)	*Abdomen*	Adrenal	Brain	Bone	Liver	Oropharynx	Lung	Lymph Node	Mesentery	Pancreas	Pelvic Region	Sub-cutaneous
MP0104	BRAF Mut	**Pembrolizumab**	**PR**	Yes (Ipilimumab)	25/08/2014	29/08/2016	105								**x**				
MP0105	BRAF WT	**Pembrolizumab**	**CR**	Yes (Ipilimumab)	10/02/2015	29/12/2017	150			**x**				**x**					**x**
MP0201	BRAF Mut	**Pembrolizumab**	**CR**	No (Radiation Therapy)	5/08/2016	27/02/2018	82				**x**	**x**		**x**					
MP0303	BRAF Mut	**Pembrolizumab**	**PR**	No	15/09/2017	Ongoing	90[a]				**x**	**x**							
MP0302	BRAF Mut	**Pembrolizumab**	**SD**	No	13/12/2017	31/05/2018	24		**x**					**x**	**x**				
MP0102	BRAF WT	**Pembrolizumab**	**PD**	No	10/12/2015	12/02/2016	9						**x**		**x**	**x**			
MP0103	BRAF Mut	**Pembrolizumab**	**PD**	No	13/05/2016	4/07/2016	7				**x**	**x**			**x**				
MP0301	BRAF WT	**Pembrolizumab**	**PD**	No	11/06/2015	18/09/2015	14												
MP0304	BRAF WT	**Pembrolizumab**	**PD**	No	03/04/2017	25/05/2017	7	**x**		**x**	**x**								

[a] Weeks in treatment as of 31/08/2019.

References

1. Calapre, L.; Giardina, T.; Robinson, C.; Reid, A.L.; Al-Ogaili, Z.; Pereira, M.R.; McEvoy, A.C.; Warburton, L.; Hayward, N.K.; Khattak, M.A., et al. Locus-specific concordance of genomic alterations between tissue and plasma circulating tumor DNA in metastatic melanoma. *Molecular oncology* **2019**, *13*, 171–184, doi:10.1002/1878-0261.12391.
2. Gray, E.S.; Rizos, H.; Reid, A.L.; Boyd, S.C.; Pereira, M.R.; Lo, J.; Tembe, V.; Freeman, J.; Lee, J.H.; Scolyer, R.A., et al. Circulating tumor DNA to monitor treatment response and detect acquired resistance in patients with metastatic melanoma. *Oncotarget* **2015**, *6*, 42008–42018, doi:10.18632/oncotarget.5788.
3. McEvoy, A.C.; Calapre, L.; Pereira, M.R.; Giardina, T.; Robinson, C.; Khattak, M.A.; Meniawy, T.M.; Pritchard, A.L.; Hayward, N.K.; Amanuel, B., et al. Sensitive droplet digital PCR method for detection of TERT promoter mutations in cell free DNA from patients with metastatic melanoma. *Oncotarget* **2017**, *8*, 78890–78900, doi:10.18632/oncotarget.20354.
4. Li, H. A statistical framework for SNP calling, mutation discovery, association mapping and population genetical parameter estimation from sequencing data. *Bioinformatics (Oxford, England)* **2011**, *27*, 2987–2993, doi:10.1093/bioinformatics/btr509.
5. Hundal, J.; Carreno, B.M.; Petti, A.A.; Linette, G.P.; Griffith, O.L.; Mardis, E.R.; Griffith, M. pVAC-Seq: A genome-guided in silico approach to identifying tumor neoantigens. *Genome medicine* **2016**, *8*, 11, doi:10.1186/s13073-016-0264-5.
6. Nielsen, M.; Lundegaard, C.; Blicher, T.; Lamberth, K.; Harndahl, M.; Justesen, S.; Roder, G.; Peters, B.; Sette, A.; Lund, O., et al. NetMHCpan, a method for quantitative predictions of peptide binding to any HLA-A and -B locus protein of known sequence. *PloS one* **2007**, *2*, e796, doi:10.1371/journal.pone.0000796.
